# 2-Chloro-*N*-(2,6-dimethyl­phen­yl)benzamide

**DOI:** 10.1107/S1600536812015607

**Published:** 2012-04-18

**Authors:** Vinola Z. Rodrigues, B. Thimme Gowda, Július Sivý, Viktor Vrábel, Jozef Kožíšek

**Affiliations:** a Department of Chemistry, Mangalore University, Mangalagangotri 574 199, Mangalore, India; b Institute of Mathematics and Physics, Faculty of Mechanical Engineering STU, Námestie Slobody 17, SK-812 37 Bratislava, Slovak Republic; c Institute of Physical Chemistry and Chemical Physics, Slovak University of Technology, Radlinského 9, SK-812 37 Bratislava, Slovak Republic

## Abstract

In the title compound, C_15_H_14_ClNO, the dihedral angle between the benzoyl and the aniline rings is 3.30 (18)°. In the crystal, N—H⋯O hydrogen bonds link the mol­ecules into chains running along the *a* axis.

## Related literature

For studies on the effects of substituents on the structures and other aspects of *N*-(ar­yl)-amides, see: Bowes *et al.* (2003 ▶); Gowda *et al.* (2000 ▶, 2007 ▶, 2008 ▶); Saeed *et al.* (2010 ▶), on *N*-chloro­aryl­sulfonamides, see: Jyothi & Gowda (2004 ▶) and on *N*-bromo­aryl­sulfonamides, see: Usha & Gowda (2006 ▶).
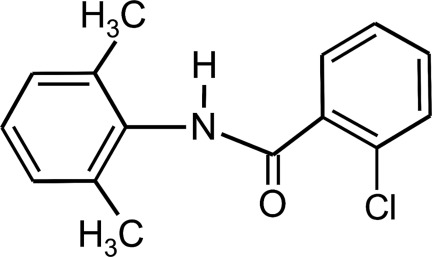


## Experimental

### 

#### Crystal data

C_15_H_14_ClNO
*M* *_r_* = 259.72
Monoclinic, 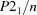

*a* = 4.8322 (3) Å
*b* = 12.7817 (10) Å
*c* = 21.8544 (12) Å
β = 90.778 (5)°
*V* = 1349.69 (15) Å^3^
*Z* = 4
Mo *K*α radiation
μ = 0.27 mm^−1^
*T* = 295 K
0.51 × 0.30 × 0.11 mm

#### Data collection

Oxford Diffraction Xcalibur Ruby Gemini diffractometer
Absorption correction: analytical [*CrysAlis RED* (Oxford Diffraction, 2009 ▶), based on expressions derived by Clark & Reid (1995 ▶)] *T*_min_ = 0.907, *T*_max_ = 0.971
18665 measured reflections
2469 independent reflections
1735 reflections with *I* > 2σ(*I*)
*R*_int_ = 0.081

#### Refinement

*R*[*F* ^2^ > 2σ(*F* ^2^)] = 0.092
*wR*(*F* ^2^) = 0.248
*S* = 1.12
2469 reflections
168 parameters
1 restraint
H atoms treated by a mixture of independent and constrained refinement
Δρ_max_ = 0.50 e Å^−3^
Δρ_min_ = −0.27 e Å^−3^

### 

Data collection: *CrysAlis CCD* (Oxford Diffraction, 2009 ▶); cell refinement: *CrysAlis CCD*; data reduction: *CrysAlis RED* (Oxford Diffraction, 2009 ▶); program(s) used to solve structure: *SHELXS97* (Sheldrick, 2008 ▶); program(s) used to refine structure: *SHELXL97* (Sheldrick, 2008 ▶); molecular graphics: *ORTEP-3* (Farrugia, 1997 ▶); software used to prepare material for publication: *SHELXL97*, *PLATON* (Spek, 2009 ▶) and *WinGX* (Farrugia, 1999 ▶).

## Supplementary Material

Crystal structure: contains datablock(s) global, I. DOI: 10.1107/S1600536812015607/bt5865sup1.cif

Structure factors: contains datablock(s) I. DOI: 10.1107/S1600536812015607/bt5865Isup2.hkl

Supplementary material file. DOI: 10.1107/S1600536812015607/bt5865Isup3.cml

Additional supplementary materials:  crystallographic information; 3D view; checkCIF report

## Figures and Tables

**Table 1:** Hydrogen-bond geometry (Å, °)

*D*—H⋯*A*	*D*—H	H⋯*A*	*D*⋯*A*	*D*—H⋯*A*
N1—H1⋯O1^i^	0.86 (2)	1.96 (2)	2.818 (4)	172 (4)

Symmetry code: (i) 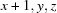.

**Fig. 1.**
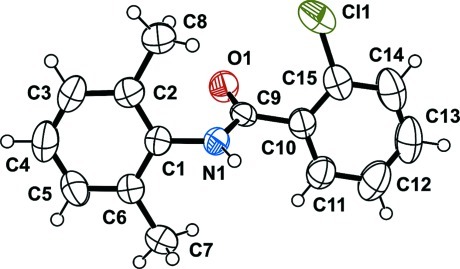
Molecular structure of the title compound with the atomic numbering scheme. Displacement ellipsoids are drawn at the 50% probability level. H atoms are represented as small spheres of arbitrary radii.

**Fig. 2.**
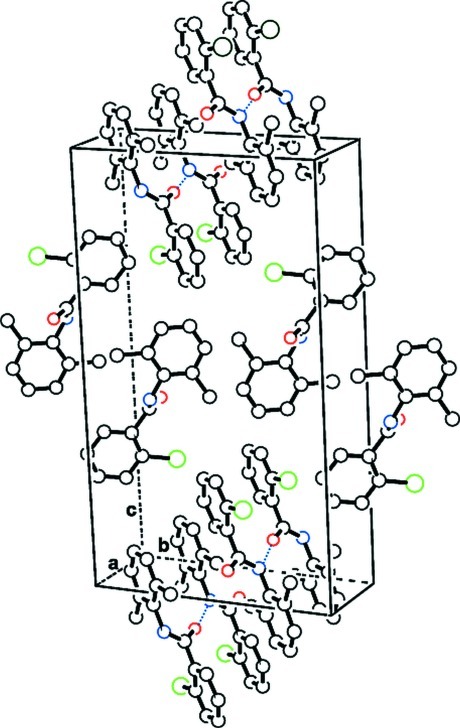
Packing view of the title compound. Molecular links along a-axis are generated by N–H···O hydrogen bonds which are shown by dashed lines. H atoms have been omitted for clarity.

## supplementary crystallographic information

### Comment

The amide and sulfonamide moieties are the constituents of many biologically important compounds. As part of studies on the substituent effects on the structures and other aspects of *N*-(aryl)-amides (Bowes *et al.*, 2003; Gowda *et al.*, 2000, 2007, 2008; Saeed *et al.*, 2010), *N*-chloroarylsulfonamides (Jyothi & Gowda, 2004) and *N*-bromoarylsulfonamides (Usha & Gowda, 2006), in the present work, the crystal structure of 2-chloro-*N*-(2,6-dimethylphenyl)benzamide has been determined (Fig. 1).

In the title compound, one of the *ortho*-methyl groups in the aniline ring is positioned *syn* to the N—H bond, while the other *ortho*- methyl group is positioned *anti* to the N—H bond, the latter and the C=O bond being *anti* to each other. Further, the amide oxygen is *syn* to the *ortho*-chloro group in the benzoyl ring, similar to that observed in 2-chloro-*N*-(2,6-dichlorophenyl)benzamide (Gowda *et al.*, 2008). In the title compound, the amide group forms dihedral angles of 63.26 (22)° and 59.88 (23)°, respectively, with the 2-chlorobenzoyl and the 2,6-dimethyl- anilino rings, while the angle between the benzoyl and the anilino rings is 3.46 (17)°.

In the crystal structure, intermolecular N1—H1···O1 hydrogen bonds (Table 1) link the molecules into infinite chains running along the *a*-axis. Part of the crystal structure is shown in Fig. 2.

### Experimental

The title compound was prepared by a method similar to the one described by Gowda *et al.* (2008). The purity of the compound was checked by determining its melting point. It was characterized by recording its infrared and NMR spectra.

Rod like colourless single crystals of the title compound used in the X-ray diffraction studies were obtained by slow evaporation of the solvent from its ethanol solution of the compound (0.5 g in about 30 ml of ethanol) at room temperature.

### Refinement

Hydrogen atoms were placed in calculated positions with C–H distances of 0.93 Å (C-aromatic), 0.96 Å (C-methyl) and constrained to ride on their parent atoms. The amide H atom was visible in a difference map and refined with the N—H distance restrained to 0.86 (1) Å. The *U*_iso_(H) values were set at 1.2*U*_eq_ (C-aromatic, N) or 1.5*U*_eq_ (C-methyl).

### Crystal data

**Table:**

C_15_H_14_ClNO	*F*(000) = 544
*M**_r_* = 259.72	*D*_x_ = 1.278 Mg m^−^^3^
Monoclinic, *P*2_1_/*n*	Mo *K*α radiation, λ = 0.71073 Å
Hall symbol: -P 2yn	Cell parameters from 3462 reflections
*a* = 4.8322 (3) Å	θ = 3.7–29.5°
*b* = 12.7817 (10) Å	µ = 0.27 mm^−^^1^
*c* = 21.8544 (12) Å	*T* = 295 K
β = 90.778 (5)°	Rod, colourless
*V* = 1349.69 (15) Å^3^	0.51 × 0.30 × 0.11 mm
*Z* = 4	

### Data collection

**Table:**

Oxford Diffraction Xcalibur Ruby Gemini diffractometer	2469 independent reflections
Graphite monochromator	1735 reflections with *I* > 2σ(*I*)
Detector resolution: 10.434 pixels mm^-1^	*R*_int_ = 0.081
ω scans	θ_max_ = 25.3°, θ_min_ = 4.2°
Absorption correction: analytical [*CrysAlis RED* (Oxford Diffraction, 2009), based on expressions derived by Clark & Reid (1995)]	*h* = −5→5
*T*_min_ = 0.907, *T*_max_ = 0.971	*k* = −15→15
18665 measured reflections	*l* = −26→26

### Refinement

**Table:**

Refinement on *F*^2^	Primary atom site location: structure-invariant direct methods
Least-squares matrix: full	Secondary atom site location: difference Fourier map
*R*[*F*^2^ > 2σ(*F*^2^)] = 0.092	Hydrogen site location: inferred from neighbouring sites
*wR*(*F*^2^) = 0.248	H atoms treated by a mixture of independent and constrained refinement
*S* = 1.12	*w* = 1/[σ^2^(*F*_o_^2^) + (0.080*P*)^2^ + 2.4052*P*] where *P* = (*F*_o_^2^ + 2*F*_c_^2^)/3
2469 reflections	(Δ/σ)_max_ < 0.001
168 parameters	Δρ_max_ = 0.50 e Å^−^^3^
1 restraint	Δρ_min_ = −0.27 e Å^−^^3^

### Special details

**Table:**

Geometry. All e.s.d.'s (except the e.s.d. in the dihedral angle between two l.s. planes) are estimated using the full covariance matrix. The cell e.s.d.'s are taken into account individually in the estimation of e.s.d.'s in distances, angles and torsion angles; correlations between e.s.d.'s in cell parameters are only used when they are defined by crystal symmetry. An approximate (isotropic) treatment of cell e.s.d.'s is used for estimating e.s.d.'s involving l.s. planes.
Refinement. Refinement of *F*^2^ against ALL reflections. The weighted *R*-factor *wR* and goodness of fit *S* are based on *F*^2^, conventional *R*-factors *R* are based on *F*, with *F* set to zero for negative *F*^2^. The threshold expression of *F*^2^ > σ(*F*^2^) is used only for calculating *R*-factors(gt) *etc*. and is not relevant to the choice of reflections for refinement. *R*-factors based on *F*^2^ are statistically about twice as large as those based on *F*, and *R*- factors based on ALL data will be even larger.

### Fractional atomic coordinates and isotropic or equivalent isotropic displacement parameters (Å^2^)

**Table:**

	*x*	*y*	*z*	*U*_iso_*/*U*_eq_	
C1	0.5223 (8)	0.2456 (4)	0.47475 (19)	0.0494 (10)	
C2	0.3612 (11)	0.3360 (4)	0.4743 (2)	0.0660 (13)	
C3	0.3033 (14)	0.3824 (5)	0.5300 (3)	0.0892 (19)	
H3	0.1927	0.4419	0.5307	0.107*	
C4	0.4038 (14)	0.3431 (5)	0.5834 (3)	0.094 (2)	
H4	0.3632	0.3761	0.6202	0.113*	
C5	0.5672 (11)	0.2539 (5)	0.5836 (2)	0.0720 (15)	
H5	0.6349	0.2271	0.6204	0.086*	
C6	0.6293 (9)	0.2050 (4)	0.5296 (2)	0.0528 (11)	
C7	0.8109 (11)	0.1086 (4)	0.5297 (2)	0.0720 (15)	
H7A	0.8096	0.0771	0.5696	0.108*	
H7B	0.9967	0.1281	0.5197	0.108*	
H7C	0.7417	0.0596	0.5	0.108*	
C8	0.2532 (14)	0.3832 (4)	0.4152 (3)	0.0830 (17)	
H8A	0.2349	0.4575	0.42	0.124*	
H8B	0.0759	0.3534	0.4052	0.124*	
H8C	0.3803	0.3685	0.3829	0.124*	
C9	0.3874 (8)	0.1464 (3)	0.38474 (18)	0.0438 (10)	
C10	0.4897 (8)	0.0847 (4)	0.3306 (2)	0.0533 (11)	
C11	0.6640 (10)	0.0015 (4)	0.3394 (2)	0.0661 (13)	
H11	0.7286	−0.0141	0.3786	0.079*	
C12	0.7455 (13)	−0.0601 (5)	0.2903 (3)	0.0911 (19)	
H12	0.8633	−0.1166	0.2968	0.109*	
C13	0.6524 (15)	−0.0373 (6)	0.2326 (3)	0.097 (2)	
H13	0.7055	−0.0789	0.1998	0.116*	
C14	0.4789 (14)	0.0476 (6)	0.2226 (3)	0.0887 (19)	
H14	0.4174	0.0643	0.1833	0.106*	
C15	0.3997 (10)	0.1063 (4)	0.2718 (2)	0.0649 (13)	
N1	0.5818 (7)	0.1927 (3)	0.41875 (15)	0.0460 (9)	
H1	0.756 (4)	0.180 (4)	0.415 (2)	0.055*	
O1	0.1424 (6)	0.1494 (3)	0.39531 (15)	0.0656 (10)	
Cl1	0.1880 (4)	0.21406 (14)	0.25687 (7)	0.0994 (7)	

### Atomic displacement parameters (Å^2^)

**Table:**

	*U*^11^	*U*^22^	*U*^33^	*U*^12^	*U*^13^	*U*^23^
C1	0.050 (2)	0.053 (3)	0.045 (2)	0.0038 (19)	0.0043 (18)	−0.0021 (19)
C2	0.079 (3)	0.063 (3)	0.057 (3)	0.019 (3)	0.001 (2)	0.000 (2)
C3	0.119 (5)	0.076 (4)	0.073 (4)	0.039 (4)	0.007 (3)	−0.022 (3)
C4	0.127 (5)	0.096 (5)	0.061 (4)	0.029 (4)	0.013 (3)	−0.028 (3)
C5	0.086 (3)	0.089 (4)	0.041 (3)	0.002 (3)	−0.001 (2)	−0.006 (3)
C6	0.056 (2)	0.056 (3)	0.046 (2)	0.004 (2)	0.0018 (19)	−0.002 (2)
C7	0.079 (3)	0.082 (4)	0.055 (3)	0.018 (3)	−0.009 (2)	0.007 (3)
C8	0.118 (5)	0.065 (3)	0.066 (3)	0.033 (3)	−0.004 (3)	0.006 (3)
C9	0.041 (2)	0.052 (3)	0.039 (2)	0.0011 (18)	−0.0020 (16)	0.0078 (18)
C10	0.042 (2)	0.070 (3)	0.048 (2)	−0.005 (2)	0.0017 (18)	−0.005 (2)
C11	0.061 (3)	0.070 (3)	0.067 (3)	0.001 (3)	0.002 (2)	−0.016 (3)
C12	0.087 (4)	0.079 (4)	0.107 (5)	0.007 (3)	0.012 (4)	−0.036 (4)
C13	0.110 (5)	0.108 (5)	0.073 (4)	−0.019 (4)	0.020 (4)	−0.046 (4)
C14	0.101 (4)	0.112 (5)	0.053 (3)	−0.019 (4)	0.000 (3)	−0.019 (3)
C15	0.068 (3)	0.074 (3)	0.052 (3)	−0.012 (3)	0.004 (2)	−0.003 (2)
N1	0.0393 (17)	0.056 (2)	0.0428 (19)	0.0052 (16)	0.0047 (15)	−0.0015 (16)
O1	0.0413 (17)	0.093 (3)	0.062 (2)	0.0015 (16)	0.0020 (14)	−0.0100 (18)
Cl1	0.1313 (14)	0.1054 (13)	0.0612 (9)	0.0210 (10)	−0.0121 (8)	0.0189 (8)

### Geometric parameters (Å, º)

**Table:**

C1—C2	1.394 (6)	C8—H8C	0.96
C1—C6	1.398 (6)	C9—O1	1.210 (5)
C1—N1	1.431 (5)	C9—N1	1.329 (5)
C2—C3	1.386 (7)	C9—C10	1.511 (6)
C2—C8	1.511 (7)	C10—C11	1.369 (7)
C3—C4	1.355 (9)	C10—C15	1.379 (7)
C3—H3	0.93	C11—C12	1.392 (8)
C4—C5	1.387 (8)	C11—H11	0.93
C4—H4	0.93	C12—C13	1.365 (10)
C5—C6	1.373 (7)	C12—H12	0.93
C5—H5	0.93	C13—C14	1.387 (9)
C6—C7	1.512 (7)	C13—H13	0.93
C7—H7A	0.96	C14—C15	1.370 (8)
C7—H7B	0.96	C14—H14	0.93
C7—H7C	0.96	C15—Cl1	1.744 (6)
C8—H8A	0.96	N1—H1	0.860 (19)
C8—H8B	0.96		
			
C2—C1—C6	120.9 (4)	C2—C8—H8C	109.5
C2—C1—N1	120.3 (4)	H8A—C8—H8C	109.5
C6—C1—N1	118.8 (4)	H8B—C8—H8C	109.5
C3—C2—C1	117.9 (5)	O1—C9—N1	124.4 (4)
C3—C2—C8	120.6 (5)	O1—C9—C10	119.8 (4)
C1—C2—C8	121.5 (4)	N1—C9—C10	115.7 (3)
C4—C3—C2	121.6 (5)	C11—C10—C15	118.2 (5)
C4—C3—H3	119.2	C11—C10—C9	120.2 (4)
C2—C3—H3	119.2	C15—C10—C9	121.6 (4)
C3—C4—C5	120.2 (5)	C10—C11—C12	120.7 (6)
C3—C4—H4	119.9	C10—C11—H11	119.6
C5—C4—H4	119.9	C12—C11—H11	119.6
C6—C5—C4	120.3 (5)	C13—C12—C11	119.9 (6)
C6—C5—H5	119.9	C13—C12—H12	120
C4—C5—H5	119.9	C11—C12—H12	120
C5—C6—C1	119.0 (4)	C12—C13—C14	120.2 (6)
C5—C6—C7	120.2 (4)	C12—C13—H13	119.9
C1—C6—C7	120.8 (4)	C14—C13—H13	119.9
C6—C7—H7A	109.5	C15—C14—C13	118.7 (6)
C6—C7—H7B	109.5	C15—C14—H14	120.7
H7A—C7—H7B	109.5	C13—C14—H14	120.7
C6—C7—H7C	109.5	C14—C15—C10	122.3 (6)
H7A—C7—H7C	109.5	C14—C15—Cl1	117.1 (5)
H7B—C7—H7C	109.5	C10—C15—Cl1	120.6 (4)
C2—C8—H8A	109.5	C9—N1—C1	122.7 (3)
C2—C8—H8B	109.5	C9—N1—H1	124 (3)
H8A—C8—H8B	109.5	C1—N1—H1	112 (3)
			
C6—C1—C2—C3	−2.0 (8)	N1—C9—C10—C15	−122.9 (5)
N1—C1—C2—C3	178.2 (5)	C15—C10—C11—C12	−0.6 (7)
C6—C1—C2—C8	177.6 (5)	C9—C10—C11—C12	175.8 (5)
N1—C1—C2—C8	−2.2 (8)	C10—C11—C12—C13	0.3 (9)
C1—C2—C3—C4	1.4 (10)	C11—C12—C13—C14	0.6 (10)
C8—C2—C3—C4	−178.2 (7)	C12—C13—C14—C15	−1.2 (10)
C2—C3—C4—C5	−0.6 (11)	C13—C14—C15—C10	0.9 (9)
C3—C4—C5—C6	0.3 (10)	C13—C14—C15—Cl1	178.6 (5)
C4—C5—C6—C1	−0.9 (8)	C11—C10—C15—C14	0.0 (7)
C4—C5—C6—C7	179.1 (5)	C9—C10—C15—C14	−176.4 (5)
C2—C1—C6—C5	1.8 (7)	C11—C10—C15—Cl1	−177.6 (4)
N1—C1—C6—C5	−178.4 (4)	C9—C10—C15—Cl1	6.0 (6)
C2—C1—C6—C7	−178.2 (5)	O1—C9—N1—C1	4.6 (7)
N1—C1—C6—C7	1.6 (7)	C10—C9—N1—C1	−173.9 (4)
O1—C9—C10—C11	−117.8 (5)	C2—C1—N1—C9	−65.8 (6)
N1—C9—C10—C11	60.8 (6)	C6—C1—N1—C9	114.4 (5)
O1—C9—C10—C15	58.5 (6)		

### Hydrogen-bond geometry (Å, º)

**Table:**

*D*—H···*A*	*D*—H	H···*A*	*D*···*A*	*D*—H···*A*
N1—H1···O1^i^	0.86 (2)	1.96 (2)	2.818 (4)	172 (4)

Symmetry code: (i) *x*+1, *y*, *z*.
